# Juvenile competitive triathlete after cardiotoxic anthracycline therapy for Acute Myeloid Leukemia

**DOI:** 10.1186/s40959-016-0016-0

**Published:** 2016-10-14

**Authors:** Pia von Korn, Manfred Vogt, Renate Oberhoffer, Peter Ewert, Jan Müller

**Affiliations:** 1 Institute of Preventive Pediatrics, Technische Universität München, Munich, Germany; 2 Department of Pediatric Cardiology and Congenital Heart Disease, Deutsches Herzzentrum München, Technische Universität München, Lazarettstr. 36, D-80636 Munich, Germany

**Keywords:** Acute myeloid leukemia, Anthracyclines, Exercise, Training, Rehabilitation

## Abstract

**Objective:**

The treatment of Acute Myeloid Leukemia (AML) leads to several functional limitations. Especially cardiac burden following cardiotoxic chemotherapy, which limits exercise and competitive sport in the long-term survivors.

**Subject and methods:**

We report on a young female amateur triathlete born in 1997, who was diagnosed with AML at the age of fifteen. She had chemotherapy with a cumulative dose of about 1000 mg/m^2^ anthracyclines and allogeneic stem cell transplantation which was successful, but she suffered from cardiotoxic systolic heart failure with a left ventricular ejection fraction (LVEF) <55 % and an impaired peak oxygen uptake of 23.2 ml/min/kg and 53 % of predicted, respectively. After medical examination and counselling with a sport scientist she started a tailored training of aerobic exercise. She was evaluated at regular intervals which resulted in increasing the training load and volume. Eventually her training hours was stepwise increased to 12 h training per week, which includes high intensity intervals.

**Results:**

Within almost 3 years, her exercise performance improved tremendously. Workload doubled from 2.1 W/kg to 4.2 W/kg, peak oxygen uptake increased from 23.2 ml/min/kg to 49.1 ml/min/kg and from 53 to 135 %, respectively. Moreover, she participated in several competitions. However, LVEF remains almost unchanged.

**Conclusion:**

With the right training and under medical surveillance competitive exercise with an anthracycline-damaged heart is still achievable. Moreover**,** competitive training and exercise seems to be safe and feasible.

## Introduction

Treatment of “Acute Myeloid Leukemia” (AML) includes chemotherapy with anthracyclines, whose cardiotoxicity is well documented in cancer literature [1]. Higher doses of anthracyclines (>250 mg/m^2^) are directly associated with an exponentially higher risk of developing cardiac pathogenesis [2, 3]. Moreover, anthracycline leads to cardiac complications like cardiomyopathy, reduced left ventricular ejection fraction (<55 %) and higher arterial stiffness in 16 % of all AML-long-term-survivors [1, 4, 5]. Since children and young adults tend to suffer from this disease a closer look towards long-term functional outcome of every single survivor has to be taken into account.

Contemporary, curative treatment of chemotherapeutic protocols, like AFM-BFM^1^, reduce the dosage of cardiotoxic anthracycline by replacing Idarubicin by L-DNR for less treatment-related mortality [6, 7]. However, studies support that also low doses of anthracyclines are still cardiotoxic [8]. Impaired cardiac response to exercise and reduced exercise capacity is frequently seen in cancer survivors treated with cardiotoxic drugs [9]. For that reason tailored exercise recommendations should be given to those patients to improve or even normalize exercise capacity [9].

Even though recent studies demonstrate comparable exercise capacity of adolescent cancer survivors and healthy controls [9] there are still many patients that suffer from exercise limitations [10]. Information regarding young competitive athlete’s after cancer therapy is still lacking.

## Case report

We report on a female amateur triathlete born in 1997 who is suffering from AML diagnosed in September 2011 at the age of 15. She received the AML-BFM 2004 chemotherapy protocol with liposomal Daunorubicin, Mitoxantron and Idarubicin as cardiotoxic anthracyclines and further allogeneic bone marrow transplantation in January 2012. Her therapeutic regime contains a dose about 750 mg per square meter body surface anthracyclines, summarized to her bodily parameters at that time multiplied with 1,43 m^2^ (cumulative dose of all given protocols: 1.072,5 mg).

Her medication nowadays consists of diuretics, Beta blocker, ACE-inhibitor and an additional hormone therapy^2^. No relapse was found since then.

**Fig. 1:**
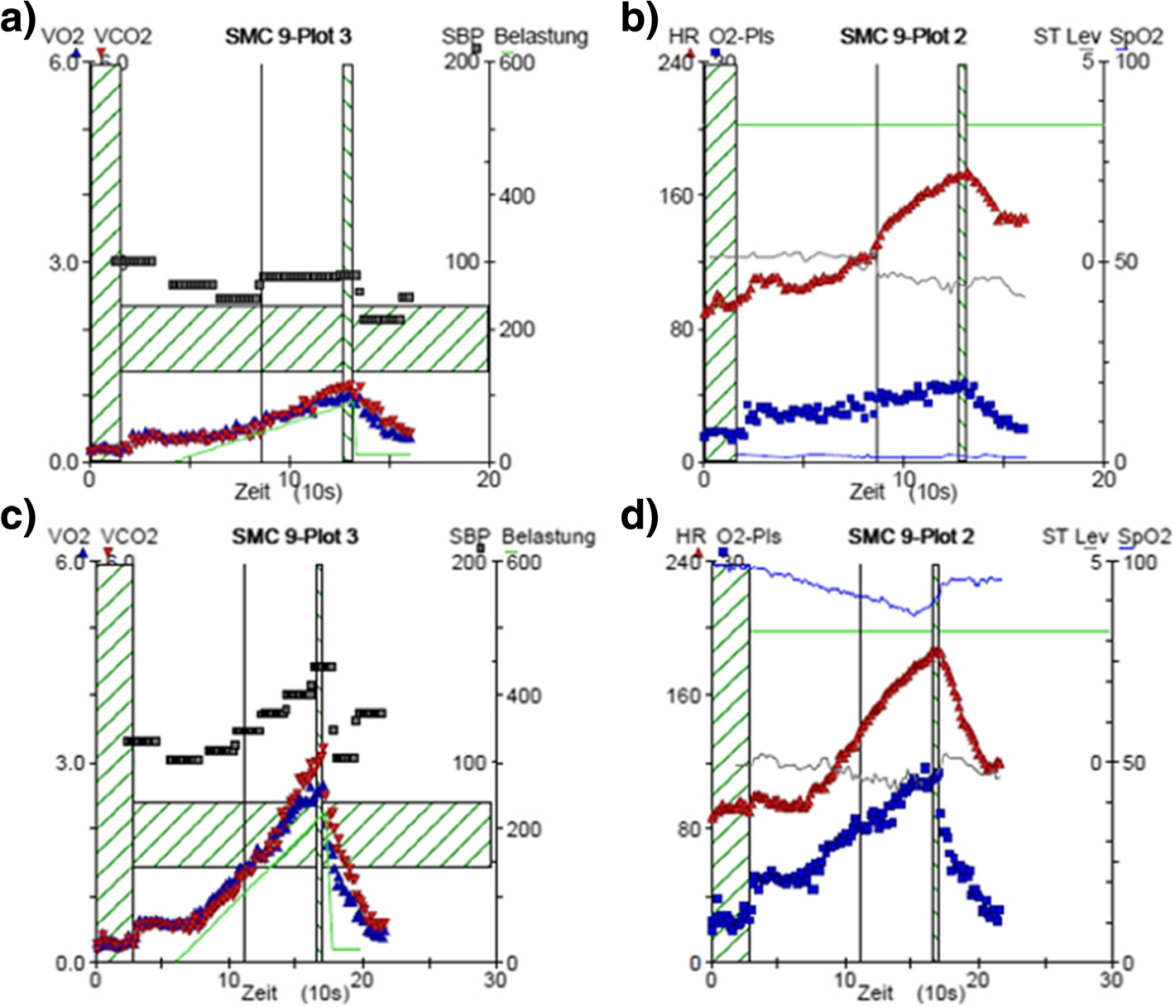
Cardiac response to exercise from cardiopulmonary exercise testing. First cardiopulmonary exercise test almost one year after therapy for Acute Myeloid Leukemia showed a drop in systolic blood pressure throughout exercise (**a**) and limitations in oxygen uptake (**a**) and heart rate (**b**). Four years after therapy for Acute Myeloid Leukemia and continuous competitive exercise training showing a tremendous improved blood pressure (**c**), oxygen and heart rate response to exercise (**d**)

In December 2012, she received her first echocardiography and the cardiopulmonary exercise test (CPET) on electronic braked bicycle ergometer at our institution. Echocardiography revealed limited contractility of the normal sizes left ventricle (Ejection fraction 50–55 %). Exercise capacity was severely reduced with a peak VO2 of 23.3 ml/min/kg and peak workload of 88 W and 2.07 W/kg, respectively. Especially the cardiac panels from the nine panel plot derived from CPET revealed a limited cardiac response to exercise (Fig. 1).

Afterwards we (athlete, sport scientist, medical doctors and parents), designed a training schedule with her coach including swimming, walking and cycling starting with just aerobic intensities. Re-evaluation was performed approximately every 6 month with the same procedures, training was adjusts, intensity and duration was progressively increased up to 12–14 h a week. Over the past three years exercise capacity increased tremendously to a peak VO2 of 49.1 ml/min/kg and peak workload of 224 W and 4.23 W/kg, respectively (Fig. 1 and Table 1). Her final CPET showed a perfect cardiac response to exercise with a well increasing VO2, heart rate and systolic blood pressure during exercise. She also competed in sprint triathlons.

In contrast her cardiac function derived from echocardiography shows only a slight improvement in contractility (Ejection fraction 55–60 %).

**Table 1:** Measures from cardiopulmonary exercise testing

	06.12.2012	06.06.2013	26.09.2013	27.03.2014	11.11.2014	29.07.2015
Resting Heart rate (beats per minute)	95	98	79	71	86	93
Peak Heart rate (beats per minute)	173	179	179	190	190	187
Resting Blood Pressure (mmHg)	100/72	85/65	99/62	93/63	83/57	110/62
Peak Blood Pressure (mmHg)	93/58	-	113/68	136/80	120/66	147/74
Peak oxygen uptake (ml/min/kg)	23.2	28.8	32.4	35.7	40.2	49.1
Peak oxygen uptake (% predicted)	53	78	82	91	107	135
Workload (Watt/kg)	2.07	2.68	3.02	3.88	3.92	4.23

## Discussion

Leisure-time physical activity is associated with reduced cancer incidence [11] and exercise interventions in adult’s cancer patients also provide positive results [12, 13]. Also competitive sports after successful cancer treatment is well documented in adults, but can be questionable, eg in the professional bicyclist Lance Armstrong.

The evidence for benefits in children is rather low. Decreased physical fitness and activity is reported in childhood cancer survivors. Unfortunately, exercise intervention studies in childhood cancer survivors were of moderate to very low quality [14].

The recent case also demonstrates a well-performed re-entry after leukemia into competitive sport, to the contrary of the majority of mostly sedentary pediatric leukemia patients [15]. Not only fighting cancer successfully, our individual developed tremendous improvements in terms of body condition and her cardiopulmonary system after therapy. Healthy cohorts showed a peak oxygen uptake of about 38.1 ml/min/kg, whereas our patient had a peak oxygen uptake of 49.1 ml/min/kg (about 30 % over average) 2 years after cancer treatment [9].

Although cardiotoxic complications were diagnosed, she started to train with low frequencies and intensities until achieving an adequate performance level from which she intensified her training with anaerobic content in high-intensity interval training (HIT). HIT is a contemporary training method not only in athletes but rather in cardiac rehabilitation [16]. HIT had shown increases in skeletal muscle oxidative capacity and endurance performance. More precisely that means a higher number and size of mitochondria, less lactate production, better utilization and energy supply during exercise and improvements in insulin sensitivity. HIT drives the metabolism much harder than regular aerobic training and adaptation in endurance and strength occur faster. Recent studies prove the beneficial effect of combined HIT and moderate training also for cancer patients [17, 18].

The structured training also led to further physiological adaptations beyond the increase in peak oxygen uptake, what substantiates findings from other investigations like improved physical and overall health benefits, immune cell recovery and body composition after hospitalization and stem cell transplantation [19, 20]. Also an appropriate blood pressure rise during exercise could be restored as well as a rise in the oxygen pulse, a surrogate for the forward stroke volume (Fig. 1a in comparison to 1b). Whereas cardiac response to exercise was extremely poor directly after therapy, in her last CPET the increase in blood pressure and oxygen pulse was very well regained over the training period of 30 months in 7 CPETs. All parameters, peak oxygen uptake, oxygen pulse and blood pressure, are predictive for survival and hospitalization in the long-term. The improvement of all means therefore a tremendous improvement in prognosis. This result, achieved with a heart harmed with a high dose of cardiotoxic anthracyclines must be seen as a pioneering success of training after cancer treatment in an adolescent.

The outline at this point is the fact that competitive sport after cancer therapy is possible only in alliance with optimal medical care, close physical examinations and tailored training schedules. When our patient reached adulthood, her body has to face the lifelong heart insufficiency medication and possible side effects and further long-term sequels could appear. However, due to medical and treatment-related sequelae, especially children and young adults have to be included in active and strengthening interventions, where they keep their physical feeling already during acute therapy [21]. Aftercare has to include widespread rehabilitation, where physical exercise and an active lifestyle has to be part of normality [22].

This case shows how cancer treatment-related toxicities and long-term sequels can be accomplished by strong will, physical capability and training effort. Moreover, it should serve as role model for all cancer survivors that suffer from heart burden and other bodily harm, whose sport and exercise was, and still is, a central part of their life.

## Abbreviations

AML: Acute Myeloid Leukemia
AMLBFM: Acute Myeloid Leukemia Berlin-Frankfurt-Münster
CPET: Cardiopulmonary exercise test
HIT: High-intensity interval training
LVEF: Left ventricular ejection fraction
VO2: Oxygen uptake

## Footnotes

1 Acute Myeloid Leukemia Berlin-Frankfurt-Münster.

2 Since 07/2012 Carvedilol (3,125 mg – 0 – 3,125 mg), Enalapril (3,75 mg – 0 – 2,5 mg), Spironothiazid (50 mg – 0 – 0). Since 06/2013 Carvedilol (3,125 mg – 0 – 3,125 mg), Digimerck pico (0,05 mg - 0–0), Spironothiazid (25 mg – 0 – 0), Lisinopril (1,25 mg – 0–0).
